# Supplementary material for: A fault-intrusion-tolerant system and deadline-aware algorithm for scheduling scientific workflow in the cloud
Source: PeerJ Comput Sci. 2021 Nov 2;7:e747. doi: 10.7717/peerj-cs.747 (PMC8576554; doi:10.7717/peerj-cs.747)
Supplement: Supplemental Information 6 [file peerj-cs-07-747-s006.zip › Myproject1.1/docs/org/cloudbus/cloudsim/HarddriveStorage.html]

HarddriveStorage


JavaScript is disabled on your browser.


- Overview
- Package
- Class
- Use
- Tree
- Deprecated
- Index
- Help

- Prev Class
- Next Class

- Frames
- No Frames

- All Classes

- Summary:
- Nested |
- Field |
- Constr |
- Method

- Detail:
- Field |
- Constr |
- Method


org.cloudbus.cloudsim

## Class HarddriveStorage

- java.lang.Object
- - org.cloudbus.cloudsim.HarddriveStorage

- All Implemented Interfaces:
  :   Storage

  Direct Known Subclasses:
  :   ClusterStorage, SanStorage

  ---

    

  ```
  public class HarddriveStorage
  extends java.lang.Object
  implements Storage
  ```

  An implementation of a storage system. It simulates the behaviour of a typical harddrive storage.
  The default values for this storage are those of a Maxtor DiamonMax 10 ATA harddisk with the
  following parameters:
  - latency = 4.17 ms- avg seek time = 9 ms- max transfer rate = 133 MB/sec

  Since:
  :   CloudSim Toolkit 1.0

- - ### Constructor Summary

    Constructors

    Constructor and Description

    `HarddriveStorage(double capacity)`

    Creates a new harddrive storage with a given capacity.

    `HarddriveStorage(java.lang.String name,
    double capacity)`

    Creates a new harddrive storage with a given name and capacity.
  - ### Method Summary

    Methods

    | Modifier and Type | Method and Description |
    | --- | --- |
    | `double` | `addFile(File file)` Adds a file to the storage. |
    | `double` | `addFile(java.util.List<File> list)` Adds a set of files to the storage. |
    | `double` | `addReservedFile(File file)` Adds a file for which the space has already been reserved. |
    | `boolean` | `contains(File file)` Checks whether a certain file is on the storage or not. |
    | `boolean` | `contains(java.lang.String fileName)` Checks whether a certain file is on the storage or not. |
    | `double` | `deleteFile(File file)` Removes a file from the storage. |
    | `File` | `deleteFile(java.lang.String fileName)` Removes a file from the storage. |
    | `double` | `deleteFile(java.lang.String fileName, File file)` Removes a file from the storage. |
    | `double` | `getAvailableSpace()` Gets the available space on this storage in MB. |
    | `double` | `getAvgSeekTime()` Gets the average seek time of the harddrive in seconds. |
    | `double` | `getCapacity()` Gets the total capacity of the storage in MB. |
    | `double` | `getCurrentSize()` Gets the current size of the stored files in MB. |
    | `File` | `getFile(java.lang.String fileName)` Gets the file with the specified name. |
    | `java.util.List<java.lang.String>` | `getFileNameList()` Gets the list of file names located on this storage. |
    | `double` | `getLatency()` Gets the latency of this harddrive in seconds. |
    | `double` | `getMaxTransferRate()` Gets the maximum transfer rate of the storage in MB/sec. |
    | `java.lang.String` | `getName()` Gets the name of the storage. |
    | `int` | `getNumStoredFile()` Gets the number of files stored on this storage. |
    | `boolean` | `hasPotentialAvailableSpace(int fileSize)` Checks whether there is enough space on the storage for a certain file. |
    | `boolean` | `isFull()` Checks if the storage is full or not. |
    | `boolean` | `renameFile(File file, java.lang.String newName)` Renames a file on the storage. |
    | `boolean` | `reserveSpace(int fileSize)` Makes a reservation of the space on the storage to store a file. |
    | `boolean` | `setAvgSeekTime(double seekTime)` Sets the average seek time of the storage in seconds. |
    | `boolean` | `setAvgSeekTime(double seekTime, ContinuousDistribution gen)` Sets the average seek time and a new generator of seek times in seconds. |
    | `boolean` | `setLatency(double latency)` Sets the latency of this harddrive in seconds. |
    | `boolean` | `setMaxTransferRate(int rate)` Sets the maximum transfer rate of this storage system in MB/sec. |

    - ### Methods inherited from class java.lang.Object

      `clone, equals, finalize, getClass, hashCode, notify, notifyAll, toString, wait, wait, wait`

- - ### Constructor Detail


    - #### HarddriveStorage

      ```
      public HarddriveStorage(java.lang.String name,
                      double capacity)
                       throws ParameterException
      ```

      Creates a new harddrive storage with a given name and capacity.

      Parameters:
      :   `name` - the name of the new harddrive storage
      :   `capacity` - the capacity in MByte

      Throws:
      :   `ParameterException` - when the name and the capacity are not valid


    - #### HarddriveStorage

      ```
      public HarddriveStorage(double capacity)
                       throws ParameterException
      ```

      Creates a new harddrive storage with a given capacity. In this case the name of the storage
      is a default name.

      Parameters:
      :   `capacity` - the capacity in MByte

      Throws:
      :   `ParameterException` - when the capacity is not valid
  - ### Method Detail


    - #### getAvailableSpace

      ```
      public double getAvailableSpace()
      ```

      Gets the available space on this storage in MB.

      **Specified by:**
      :   `getAvailableSpace` in interface `Storage`

      Returns:
      :   the available space in MB


    - #### isFull

      ```
      public boolean isFull()
      ```

      Checks if the storage is full or not.

      **Specified by:**
      :   `isFull` in interface `Storage`

      Returns:
      :   true if the storage is full, false otherwise


    - #### getNumStoredFile

      ```
      public int getNumStoredFile()
      ```

      Gets the number of files stored on this storage.

      **Specified by:**
      :   `getNumStoredFile` in interface `Storage`

      Returns:
      :   the number of stored files


    - #### reserveSpace

      ```
      public boolean reserveSpace(int fileSize)
      ```

      Makes a reservation of the space on the storage to store a file.

      **Specified by:**
      :   `reserveSpace` in interface `Storage`

      Parameters:
      :   `fileSize` - the size to be reserved in MB

      Returns:
      :   true if reservation succeeded, false otherwise


    - #### addReservedFile

      ```
      public double addReservedFile(File file)
      ```

      Adds a file for which the space has already been reserved. The time taken (in seconds) for
      adding the file can also be found using `gridsim.datagrid.File#getTransactionTime()`.

      **Specified by:**
      :   `addReservedFile` in interface `Storage`

      Parameters:
      :   `file` - the file to be added

      Returns:
      :   the time (in seconds) required to add the file


    - #### hasPotentialAvailableSpace

      ```
      public boolean hasPotentialAvailableSpace(int fileSize)
      ```

      Checks whether there is enough space on the storage for a certain file.

      **Specified by:**
      :   `hasPotentialAvailableSpace` in interface `Storage`

      Parameters:
      :   `fileSize` - a FileAttribute object to compare to

      Returns:
      :   true if enough space available, false otherwise


    - #### getCapacity

      ```
      public double getCapacity()
      ```

      Gets the total capacity of the storage in MB.

      **Specified by:**
      :   `getCapacity` in interface `Storage`

      Returns:
      :   the capacity of the storage in MB


    - #### getCurrentSize

      ```
      public double getCurrentSize()
      ```

      Gets the current size of the stored files in MB.

      **Specified by:**
      :   `getCurrentSize` in interface `Storage`

      Returns:
      :   the current size of the stored files in MB


    - #### getName

      ```
      public java.lang.String getName()
      ```

      Gets the name of the storage.

      **Specified by:**
      :   `getName` in interface `Storage`

      Returns:
      :   the name of this storage


    - #### setLatency

      ```
      public boolean setLatency(double latency)
      ```

      Sets the latency of this harddrive in seconds.

      Parameters:
      :   `latency` - the new latency in seconds

      Returns:
      :   true if the setting succeeded, false otherwise


    - #### getLatency

      ```
      public double getLatency()
      ```

      Gets the latency of this harddrive in seconds.

      Returns:
      :   the latency in seconds


    - #### setMaxTransferRate

      ```
      public boolean setMaxTransferRate(int rate)
      ```

      Sets the maximum transfer rate of this storage system in MB/sec.

      **Specified by:**
      :   `setMaxTransferRate` in interface `Storage`

      Parameters:
      :   `rate` - the maximum transfer rate in MB/sec

      Returns:
      :   true if the setting succeeded, false otherwise


    - #### getMaxTransferRate

      ```
      public double getMaxTransferRate()
      ```

      Gets the maximum transfer rate of the storage in MB/sec.

      **Specified by:**
      :   `getMaxTransferRate` in interface `Storage`

      Returns:
      :   the maximum transfer rate in MB/sec


    - #### setAvgSeekTime

      ```
      public boolean setAvgSeekTime(double seekTime)
      ```

      Sets the average seek time of the storage in seconds.

      Parameters:
      :   `seekTime` - the average seek time in seconds

      Returns:
      :   true if the setting succeeded, false otherwise


    - #### setAvgSeekTime

      ```
      public boolean setAvgSeekTime(double seekTime,
                           ContinuousDistribution gen)
      ```

      Sets the average seek time and a new generator of seek times in seconds. The generator
      determines a randomized seek time.

      Parameters:
      :   `seekTime` - the average seek time in seconds
      :   `gen` - the ContinuousGenerator which generates seek times

      Returns:
      :   true if the setting succeeded, false otherwise


    - #### getAvgSeekTime

      ```
      public double getAvgSeekTime()
      ```

      Gets the average seek time of the harddrive in seconds.

      Returns:
      :   the average seek time in seconds


    - #### getFile

      ```
      public File getFile(java.lang.String fileName)
      ```

      Gets the file with the specified name. The time taken (in seconds) for getting the file can
      also be found using `gridsim.datagrid.File#getTransactionTime()`.

      **Specified by:**
      :   `getFile` in interface `Storage`

      Parameters:
      :   `fileName` - the name of the needed file

      Returns:
      :   the file with the specified filename


    - #### getFileNameList

      ```
      public java.util.List<java.lang.String> getFileNameList()
      ```

      Gets the list of file names located on this storage.

      **Specified by:**
      :   `getFileNameList` in interface `Storage`

      Returns:
      :   a List of file names


    - #### addFile

      ```
      public double addFile(File file)
      ```

      Adds a file to the storage. First, the method checks if there is enough space on the storage,
      then it checks if the file with the same name is already taken to avoid duplicate filenames.   
      The time taken (in seconds) for adding the file can also be found using
      `gridsim.datagrid.File#getTransactionTime()`.

      **Specified by:**
      :   `addFile` in interface `Storage`

      Parameters:
      :   `file` - the file to be added

      Returns:
      :   the time taken (in seconds) for adding the specified file


    - #### addFile

      ```
      public double addFile(java.util.List<File> list)
      ```

      Adds a set of files to the storage. Runs through the list of files and save all of them. The
      time taken (in seconds) for adding each file can also be found using
      `gridsim.datagrid.File#getTransactionTime()`.

      **Specified by:**
      :   `addFile` in interface `Storage`

      Parameters:
      :   `list` - the files to be added

      Returns:
      :   the time taken (in seconds) for adding the specified files


    - #### deleteFile

      ```
      public File deleteFile(java.lang.String fileName)
      ```

      Removes a file from the storage. The time taken (in seconds) for deleting the file can also
      be found using `gridsim.datagrid.File#getTransactionTime()`.

      **Specified by:**
      :   `deleteFile` in interface `Storage`

      Parameters:
      :   `fileName` - the name of the file to be removed

      Returns:
      :   the deleted file


    - #### deleteFile

      ```
      public double deleteFile(java.lang.String fileName,
                      File file)
      ```

      Removes a file from the storage. The time taken (in seconds) for deleting the file can also
      be found using `gridsim.datagrid.File#getTransactionTime()`.

      **Specified by:**
      :   `deleteFile` in interface `Storage`

      Parameters:
      :   `fileName` - the name of the file to be removed
      :   `file` - the file which is removed from the storage is returned through this parameter

      Returns:
      :   the time taken (in seconds) for deleting the specified file


    - #### deleteFile

      ```
      public double deleteFile(File file)
      ```

      Removes a file from the storage. The time taken (in seconds) for deleting the file can also
      be found using `gridsim.datagrid.File#getTransactionTime()`.

      **Specified by:**
      :   `deleteFile` in interface `Storage`

      Parameters:
      :   `file` - the file which is removed from the storage is returned through this parameter

      Returns:
      :   the time taken (in seconds) for deleting the specified file


    - #### contains

      ```
      public boolean contains(java.lang.String fileName)
      ```

      Checks whether a certain file is on the storage or not.

      **Specified by:**
      :   `contains` in interface `Storage`

      Parameters:
      :   `fileName` - the name of the file we are looking for

      Returns:
      :   true if the file is in the storage, false otherwise


    - #### contains

      ```
      public boolean contains(File file)
      ```

      Checks whether a certain file is on the storage or not.

      **Specified by:**
      :   `contains` in interface `Storage`

      Parameters:
      :   `file` - the file we are looking for

      Returns:
      :   true if the file is in the storage, false otherwise


    - #### renameFile

      ```
      public boolean renameFile(File file,
                       java.lang.String newName)
      ```

      Renames a file on the storage. The time taken (in seconds) for renaming the file can also be
      found using `gridsim.datagrid.File#getTransactionTime()`.

      **Specified by:**
      :   `renameFile` in interface `Storage`

      Parameters:
      :   `file` - the file we would like to rename
      :   `newName` - the new name of the file

      Returns:
      :   true if the renaming succeeded, false otherwise


- Overview
- Package
- Class
- Use
- Tree
- Deprecated
- Index
- Help

- Prev Class
- Next Class

- Frames
- No Frames

- All Classes

- Summary:
- Nested |
- Field |
- Constr |
- Method

- Detail:
- Field |
- Constr |
- Method
